# Association of cognitive impairment with the interaction between chronic kidney disease and depression: findings from NHANES 2011–2014

**DOI:** 10.1186/s12888-024-05769-1

**Published:** 2024-04-24

**Authors:** Tong Zhou, Jiayu Zhao, Yimei Ma, Linqian He, Zhouting Ren, Kun Yang, Jincheng Tang, Jiali Liu, Jiaming Luo, Heping Zhang

**Affiliations:** 1 Department of Nephrology, Affiliated Hospital of North Sichuan Medical College, 1 Maoyuan Road, Nanchong city, Sichuan Province 637000 China; 2 Department of physician, Nanchong Psychosomatic Hospital, Nanchong, China; 3 Department of Clinical Medicine, North Sichuan Medical University, Nanchong, China; 4 Mental Health Center, Affiliated Hospital of North Sichuan Medical College, Nanchong, China; 5 School of Psychiatry, North Sichuan Medical College, Nanchong, China

**Keywords:** Cognitive impairment, Chronic kidney disease, Depression, Interaction

## Abstract

**Background:**

Cognitive impairment (CoI), chronic kidney disease (CKD), and depression are prevalent among older adults and are interrelated, imposing a significant disease burden. This study evaluates the association of CKD and depression with CoI and explores their potential interactions.

**Method:**

Data for this study were sourced from the 2011–2014 National Health and Nutritional Examination Survey (NHANES). Multiple binary logistic regression models assessed the relationship between CKD, depression, and CoI while controlling for confounders. The interactions were measured using the relative excess risk of interaction (RERI), the attributable proportion of interaction (AP), and the synergy index (S).

**Results:**

A total of 2,666 participants (weighted *n* = 49,251,515) were included in the study, of which 700 (16.00%) had CoI. After adjusting for confounding factors, the risk of CoI was higher in patients with CKD compared to non-CKD participants (odds ratio [OR] = 1.49, 95% confidence interval [CI]:1.12–1.99). The risk of CoI was significantly increased in patients with depression compared to those without (OR = 2.29, 95% CI: 1.73–3.03). Furthermore, there was a significant additive interaction between CKD and depression in terms of the increased risk of CoI (adjusted RERI = 2.01, [95% CI: 0.31–3.71], adjusted AP = 0.50 [95% CI: 0.25–0.75], adjusted S = 2.97 [95% CI: 1.27–6.92]).

**Conclusion:**

CKD and depression synergistically affect CoI, particularly when moderate-to-severe depression co-occurs with CKD. Clinicians should be mindful of the combined impact on patients with CoI. Further research is needed to elucidate the underlying mechanisms and assess the effects specific to different CKD stages.

**Supplementary Information:**

The online version contains supplementary material available at 10.1186/s12888-024-05769-1.

## Introduction

Cognitive impairment (CoI) is characterized by a decline in one or more brain functions, such as memory, learning, attention, decision-making, and executive abilities, ranging in severity from mild to severe [1]. The incidence of CoI has been increasing globally, particularly among individuals aged ≥ 65 years, where the prevalence of mild cognitive impairment (MCI) ranges from 10 to 20% and increases with age [2]. Severe CoI frequently leads to a diagnosis of dementia, the fifth leading cause of death globally. It significantly impairs the patient’s ability to care for themselves, imposing a heavy disease burden [3]. The prevalence of CoI in the United States has been rising, with rates increasing from 6.0% in 1997 to 7.1% in 2018 [4]. The healthcare cost for individuals with low cognitive function has exceeded 300 billion dollars, creating a substantial economic burden on patients and society [5]. Therefore, it is crucial to identify the reversible risk factors for CoI and their interactions to implement appropriate interventions, reduce the incidence of CoI, and improve the quality of life for the affected individuals.

Chronic kidney disease (CKD) is a prevalent and serious global health problem that directly contributes to morbidity and mortality, and is a significant risk factor for cardiovascular disease [6]. In advanced stages, CKD frequently requires life-sustaining treatments, such as dialysis. The prevalence of CKD in the general population exceeds 10% and can be even greater than 50% in high-risk populations [7], particularly among older adults. Emerging studies have revealed a strong association between CKD and a high prevalence of CoI [8], with cognitive function declining at all stages of CKD [9]. In patients undergoing hemodialysis, the prevalence of CoI is 75%, affecting all cognitive domains [10]. Studies have demonstrated a close relationship between the decline in renal function and brain function, resulting in poor neuropsychological performance, especially in individuals with an estimated glomerular filtration rate (eGFR) < 45 mL/min/1.73 m^2^ and those aged 60–70 years [11]. A causality study based on genome-wide association study (GWAS) data has indicated that the deterioration of kidney function contributes to the decline of brain functions in multiple regions, leading to CoI [10]. Moreover, CKD is one of the strongest risk factors for MCI and dementia, surpassed only by stroke and long-term use of anti-anxiety drugs [12, 13]. Considering the negative impact of CKD on cognitive function, it is crucial to study the association between CKD and its related complications with CoI.

Depression is a prevalent mental disorder, and ranks as the third leading cause of the global burden of mental illness [14]. From 1990 to 2016, major depressive disorder ranked the second cause of US Years Lived With Disability (YLDs) [15]. Individuals with depression often experience low mood, and loss of interest and pleasure, leading to poor health and decreased work capacity, resulting in substantial losses for patients and the global economy [16]. Studies have demonstrated an association between depressive symptoms, particularly lack of pleasure and negative emotions, and cognitive decline in older African Americans [17]. Another multiethnic study found that, depression independently predicted a faster progression to incident cognitive impairment, compared with non-Hispanic Whites, Hispanics and Asian participants had a higher hazard for progression, and previously established risk factors between depression and dementia were not found among AA and nHW participants [18].Furthermore, depressive symptoms are linked to lower scores across various aspects of cognitive function. Despite adjusting for confounders, depressive symptoms remain significantly associated with lower scores in memory, language, and processing speed [19]. Severe depression frequently manifests with CoI as a prominent feature, and despite alleviating depression symptoms, individuals experience substantial CoI, including memory, executive function, and attention [20]. In summary, depression is highly prevalent among older people, the point prevalence of depression in late life was 13.2% [21], and depressive symptoms are closely associated with cognitive decline [22]. Patients with depression have a higher incidence of CoI [23, 24] and face an increased risk of dementia [25]. Furthermore, there is a strong association between CKD and depression. In the United States, the prevalence of depression in adults with CKD is approximately 10–20% [26, 27]. The coexistence of CKD and depression is associated with a higher risk of hospitalization and death [27], leading to a further decline in the quality of life of patients [28].

While previous research has extensively reported CKD and depression as independent risk factors for CoI, the interaction between CKD and depression concerning CoI has not been widely studied. We hypothesize that when CKD, depression, and CoI coexist, there could be distinct risk combinations, such that the presence of both CKD and depression significantly increases the risk of CoI. We used relevant data from the National Health and Nutrition Examination Survey (NHANES) to test this hypothesis and investigate the interaction between depression and CKD on CoI. If an interactive effect is observed, indicating a change in the risk of CoI when CKD and depression coexist, it would suggest the importance for clinicians to identify the presence of depression and CKD in patients with CoI. This finding could provide practical implications and guidance to clinical doctors when deciding appropriate intervention measures for patients with CoI.

## Methods

### Study population

The study utilizes data from NHANES, a comprehensive nationwide cross-sectional study conducted by the National Center for Health Statistics (NCHS), to assess the nutrition and health of individuals in the United States. NHANES employs complex multi-stage probability sampling methods and examines approximately 5,000 individuals annually, providing nationally representative data in two-year cycles. We analyzed publicly available data from NHANES 2011–2014, which included cognitive test data for individuals ≥ 60 years. The NCHS Ethics Review Board approved the survey, and all participants provided written informed consent.

**Fig. 1:**
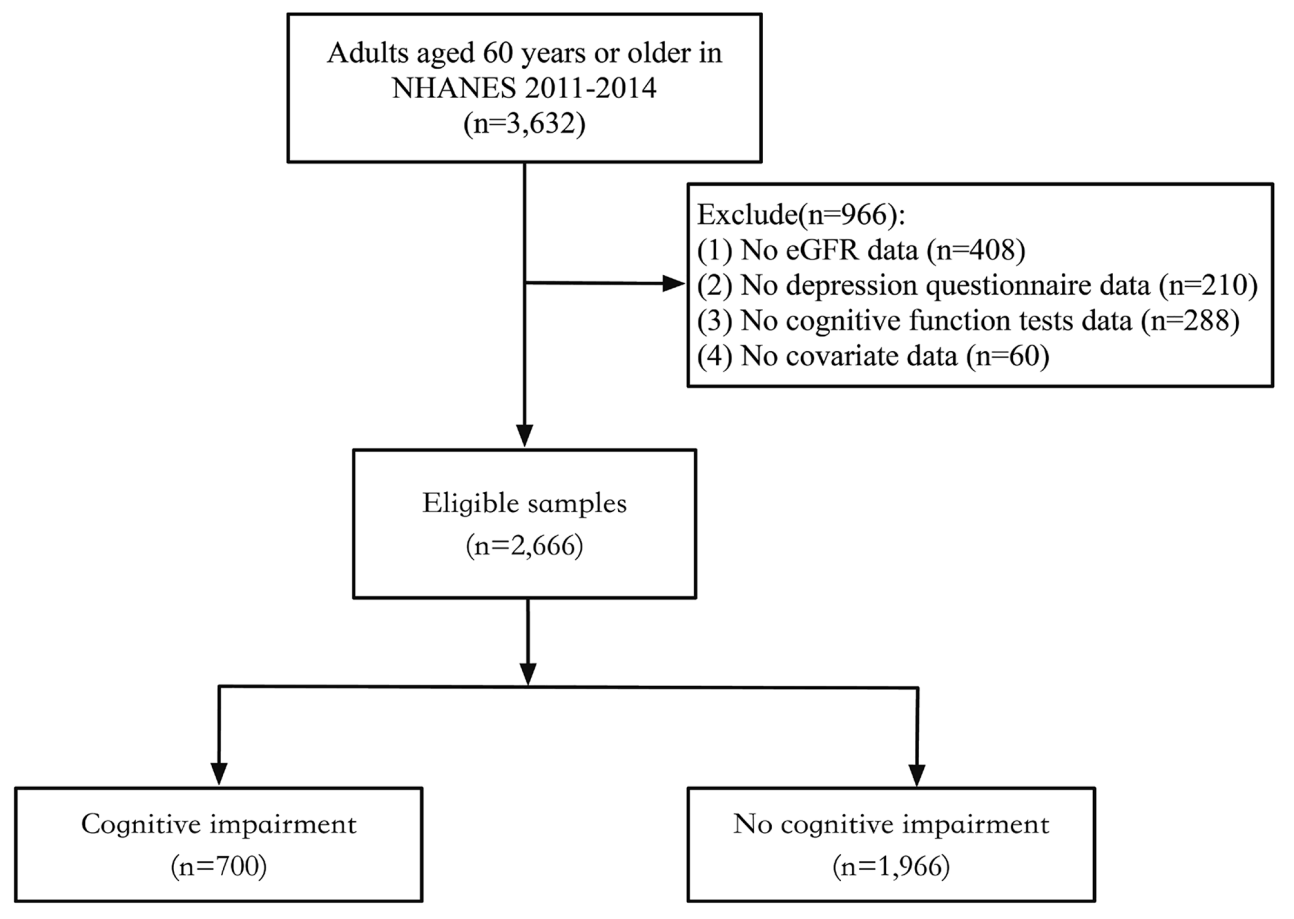
Flowchart of participants in this study

From the NHANES database, 3,632 participants were extracted. After excluding those with missing data for eGFR (*n* = 408), depression questionnaire (*n* = 210), cognitive function tests (*n* = 288), and other covariates (*n* = 60), a final sample of 2,666 participants was included in this study. The participant selection flowchart is presented in Fig. 1.

### Outcome variable

The outcome variable of interest is the presence of CoI in the study participants. NHANES assesses cognitive function through face-to-face interviews conducted by trained interviewers at mobile examination centers. The assessment includes three tests: the Consortium to Establish a Registry for Alzheimer’s Disease (CERAD) word learning and recall module, the animal fluency test (AFT), and the digit symbol substitution test (DSST). These tests are widely used for large-scale CoI screening and epidemiological research [29–31]. The CERAD Word Learning (CERAD W-L) test evaluates immediate and delayed learning abilities related to new linguistic information. It is primarily composed of three consecutive learning trials and a delayed recall. The AFT measures categorical verbal fluency, a component of executive function. The DSST, a subtest of the Wechsler Adult Intelligence Scale (WAIS-III), evaluates processing speed, sustained attention, and working memory. Higher scores in all three tests indicate better cognitive functioning. There is no gold standard threshold for CoI in these tests; hence, we followed the methodology used in previous studies [32, 33]. We defined CoI as the lowest 25th percentile of total scores across the three tests in our study cohort.

### Explanatory variables

This study employed the Jaffe rate method (kinetic alkaline picrate) to measure serum creatinine concentrations and utilized isotope dilution mass spectrometry (IDMS) for creatinine calibration. The eGFR was calculated using the Chronic Kidney Disease Epidemiology Collaboration (CKD-EPI) equation [34]. CKD was defined as eGFR < 60 mL/min/1.73 m^2^, with eGFR < 30 mL/min/1.73 m^2^ indicating progressive CKD. Depression was measured using the Patient Health Questionnaire (PHQ-9), a nine-item screening tool that assesses the frequency of depressive symptoms over the past two weeks. PHQ-9 scores range from 0 to 27, with 0–4 indicating no depression, 5–9 indicating mild depression, 10–14 denoting moderate depression, and ≥ 15 indicating severe depression [35].

### Covariates

Potential confounding factors influencing cognitive function were collected, including sociodemographic information, lifestyle habits, and medical history. Sociodemographic data (age, sex, race, education, annual family income, marital status), lifestyle habits (work activity, smoking status, alcohol user), and medical history (hypertension, diabetes, cardiovascular disease, sleep disorders) were obtained through structured questionnaires administered during home interviews. Work activity intensity was assessed by enquiring, “*Does your work involve intense/moderate/light activity*?” Participants were categorized as smokers if they answered “yes” to the question, “*Have you smoked at least 100 cigarettes in your lifetime?*” Similarly, participants were categorized as drinkers if they responded affirmatively to the question, “I*n any given year, have you consumed at least 12 drinks of any kind of alcoholic beverage?*” Diseases were diagnosed by asking participants, “*Have you been told by a doctor/health professional that you have hypertension/diabetes/cardiovascular disease/sleep disorders?*” The term “cardiovascular disease” includes congestive heart failure, coronary heart disease, angina, heart attack, and stroke.

### Statistical analysis

Data extraction and analysis were performed using R software (version 4.2.2). The Mobile Examination Center (MEC) exam weights served as the weight variable for this study. The normally distributed continuous variables were presented as mean (standard deviation) after weighting, and group comparisons were performed using weighted t-tests. Categorical variables were described by case numbers and weighted prevalence (n[weighted%]), with group comparisons analyzed using chi-square tests. Binary logistic regression analysis was employed to construct models. Model 1 was a crude model that did not adjust for confounding factors. Model 2 adjusted for age and race, while Model 3, in addition to the variables adjusted in Model 2, also adjusted for education, annual family income, BMI, marital status, work activity, alcohol user, hypertension, diabetes, and cardiovascular diseases. Furthermore, additive models were constructed to investigate potential interactions. The additive interaction between CKD and depression regarding CoI was assessed based on whether the estimated combined effect of the two factors exceeded the sum of their independent impacts. The presence of an additive interaction was evaluated using the Relative Excess Risk due to Interaction (RERI), Attributable Proportion due to Interaction (AP), and Synergy Index (S). The presence of an additive interaction was indicated if the confidence intervals for RERI and AP did not include 0 and those of S did not include 1. Subgroup analyses were performed on variables including the depression severity, CKD severity, sex, and BMI. Statistical significance was defined as two-tailed p-value < 0.05.

## Results

### Description of study participants

A total of 2,666 participants (weighted *n* = 49,251,515) were included in this study, with an average age of 69 years and an average BMI of 29.10 kg/m^2^. Among the participants, 54.07% (*n* = 1,363) were female. Non-Hispanic white participants accounted for 80.33% (*n* = 1,304), followed by non-Hispanic Black (*n* = 603, 7.83%), Hispanic (*n* = 275, 3.66%), Mexican American (*n* = 234, 3.33%), Asian (*n* = 214, 3.21%), and other races (*n* = 36, 1.64%). The average eGFR was 73 mL/min/1.73 m^2^; 22.00% (*n* = 604) had CKD, with 1.71% (*n* = 59) in the progressive stage of CKD. Among the participants, 21.12% experienced depression, with 13.67% classified as having mild depression and 7.45% as having moderate-to-severe depression. Additionally, 700 participants (*n* = 16.00%) exhibited CoI. The characteristics of the study population are presented in Table 1.

### Distribution of population with and without CoI

Table 1 reveals that patients with CoI were significantly older (73 years vs. 68 years, *P* < 0.001) than those without CoI. Moreover, significant differences were observed between the CoI and non-CoI groups concerning race, education, annual family income, marital status, work activity, alcohol user, hypertension, diabetes, cardiovascular disease, CKD, and depression (*P* < 0.01).

**Table 1:** Characteristics of participants

		Cognitive Function		
Characteristics	Overall, *N* = 2,666	No CoI *N* = 1,966 (84%)	CoI *N* = 700 (16%)	P-value
**Age, years** ^**a**^	69(7.00)	68(6.00)	73(7.00)	< **0.001**
**Sex, n (%)** ^**b**^				0.5
Female	1,363(54.07)	1,051(54.43)	312(52.12)	
Male	1,303(46.93)	915(45.57)	388(47.88)	
**Race, n (%)** ^**b**^				**< 0.001**
Mexican American	234(3.33)	142(2.46)	89(8.00)	
Non-Hispanic White	1,304(80.33)	1,079(84.19)	225(59.53)	
Non-Hispanic Black	603(7.83)	388(6.05)	215(17.41)	
Hispanic	275(3.66)	145(2.25)	130(11.27)	
Asian	214(3.21)	179(3.20)	35(3.24)	
Other	36(1.64)	30(1.84)	6(0.55)	
**Education, n (%)** ^**b**^				< **0.001**
Less than high school	659(15.67)	283(10.44)	376(43.86)	
High school graduate or GED	627(21.92)	464(21.11)	163(26.30)	
College or above	1,380(62.40)	1,219(68.45)	161(29.85)	
**Annual family income, n (%)** ^**b**^				< **0.001**
< $20,000	669(16.54)	389(13.07)	280(35.22)	
≥ $20,000	1,997(83.46)	1,577(86.93)	420(64.78)	
**Marital status, n (%)** ^**b**^				< **0.001**
Never married	153(4.38)	113(4.14)	40(5.64)	
Married/ with a partner	1,554(65.04)	1,206(67.67)	348(50.86)	
Widowed	510(16.60)	319(14.30)	191(28.98)	
Divorced or separated	449(13.99)	328(13.89)	121(14.52)	
**Work activity, n (%)** ^**b**^				< **0.001**
No or lower	1,843(64.59)	1,297(62.11)	546(77.90)	
Moderate	526(22.13)	422(23.35)	104(15.57)	
Vigorous	297(13.28)	247(15.54)	50(6.53)	
**BMI**^**a**^, **kg/m**^**2**^	29.10(6.30)	29.10(6.20)	28.70(6.50)	0.15
**Smoker, n (%)** ^**b**^	1,349(50.01)	979(49.60)	370(52.21)	0.4
**Alcohol user, n (%)** ^**b**^	1,830(72.87)	1,398(75.17)	432(60.50)	< **0.001**
**Hypertension, n (%)** ^**b**^	1,657(58.28)	1,176(56.10)	481(70.00)	< **0.001**
**Diabetes, n (%)** ^**b**^	610(19.00)	399(17.48)	211(27.21)	**0.003**
**CVD, n (%)** ^**b**^	576(21.47)	364(19.09)	212(34.32)	< **0.001**
**Sleep disorder, n (%)** ^**b**^	317(11.97)	244(12.31)	73(10.13)	0.2
**eGFR**^**a**^, **mL/min/1.73 m**^**2**^	73(18.00)	75(17.00)	66(22.00)	< **0.001**
**CKD, n (%)** ^**b**^	604(22.42)	384(19.39)	220(38.72)	**< 0.001**
**Depression, n (%)** ^**b**^	660(21.12)	413(18.36)	247(36.01)	< **0.001**

^a^Mean(Standard deviation). ^b^ Unweigthed frequency counts and weigthed percentages. Abbreviations: BMI, Body mass index; CVD, Cardiovascular disease; eGFR, estimated glomerular filtration rate; CKD, Chronic kidney disease; CoI, cognitive impairment; GED, General Educational Development

### Association between CKD and CoI

**Fig. 2:**
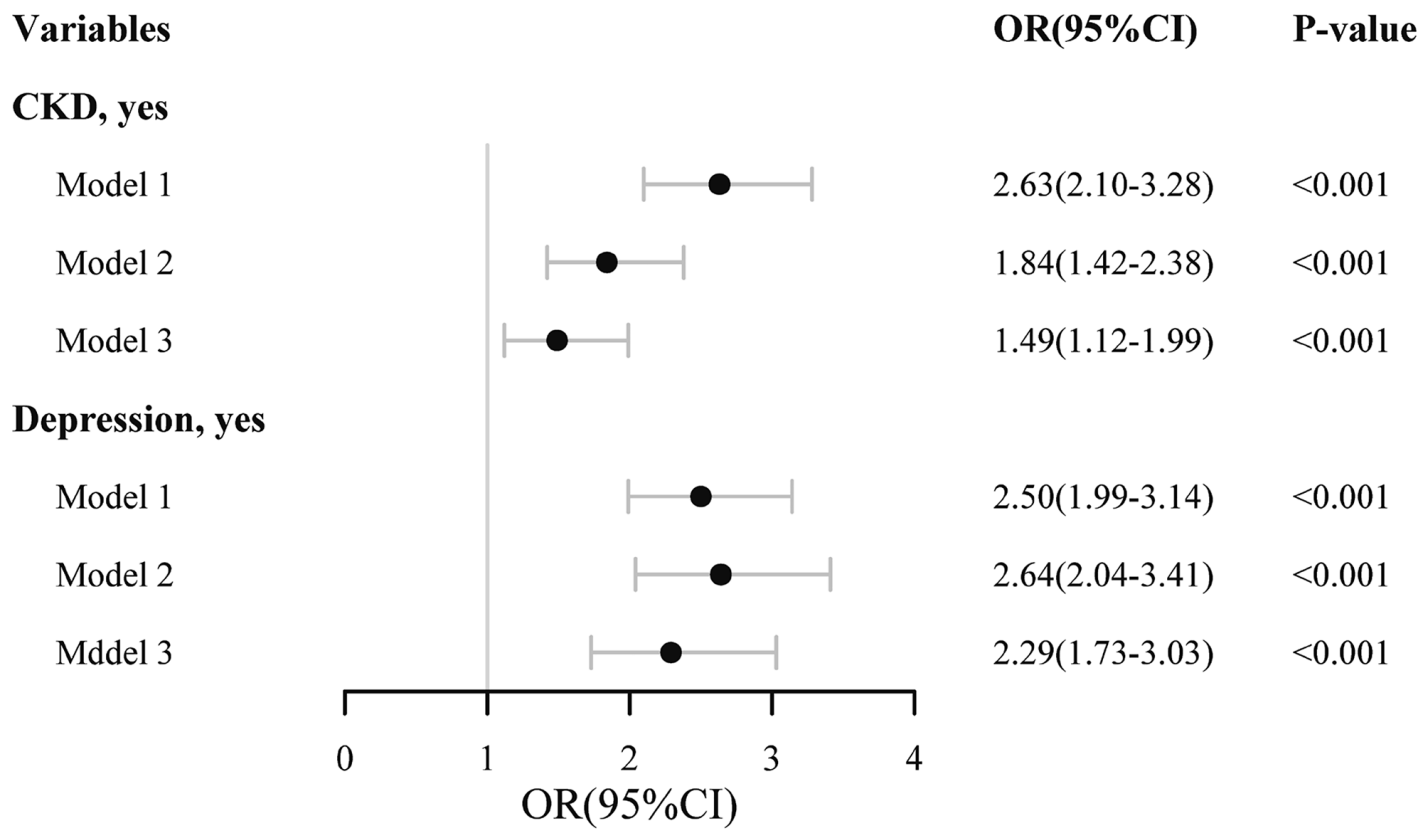
Multivariate logistic regression of CKD and depression for participants with cognitive impairment. (Model 1, unadjusted model; Model 2, adjustment for age, race; Model 3, adjustment for age, race, education, annual family income, BMI, marital status, work activity, alcohol user, hypertention, diabetes, CVD. Abbreviations: CKD, chronic kidney disease; OR, odds ratio; CI, confidence interval; CVD, cardiovascular diseases)

Compared to participants without CKD, those with CKD exhibited a significantly higher risk of CoI. Model 1 yielded a crude odds ratio (OR) of 2.63 (with a 95% confidence interval [CI] of 2.10–3.28); in Model 2, the OR was 1.84 (95% CI: 1.42–2.38) (*P* < 0.001). In confounder-adjusted Model 3, patients with CKD had a higher risk of CoI compared to non-CKD patients (OR = 1.49 [95% CI: 1.12–1.99], *P* < 0.001). The relationship between CKD and CoI is illustrated in Fig. 2.

### Association between depression and CoI

In Model 3, individuals with depression had a positive correlation with the risk of having CoI compared to those without depression (OR: 2.29 [95% CI: 1.73–3.03], *P* < 0.001). The positive correlation between depression and CoI is depicted in Fig. 2.

### Effect of the interaction between CKD and depression on CoI

**Fig. 3:**
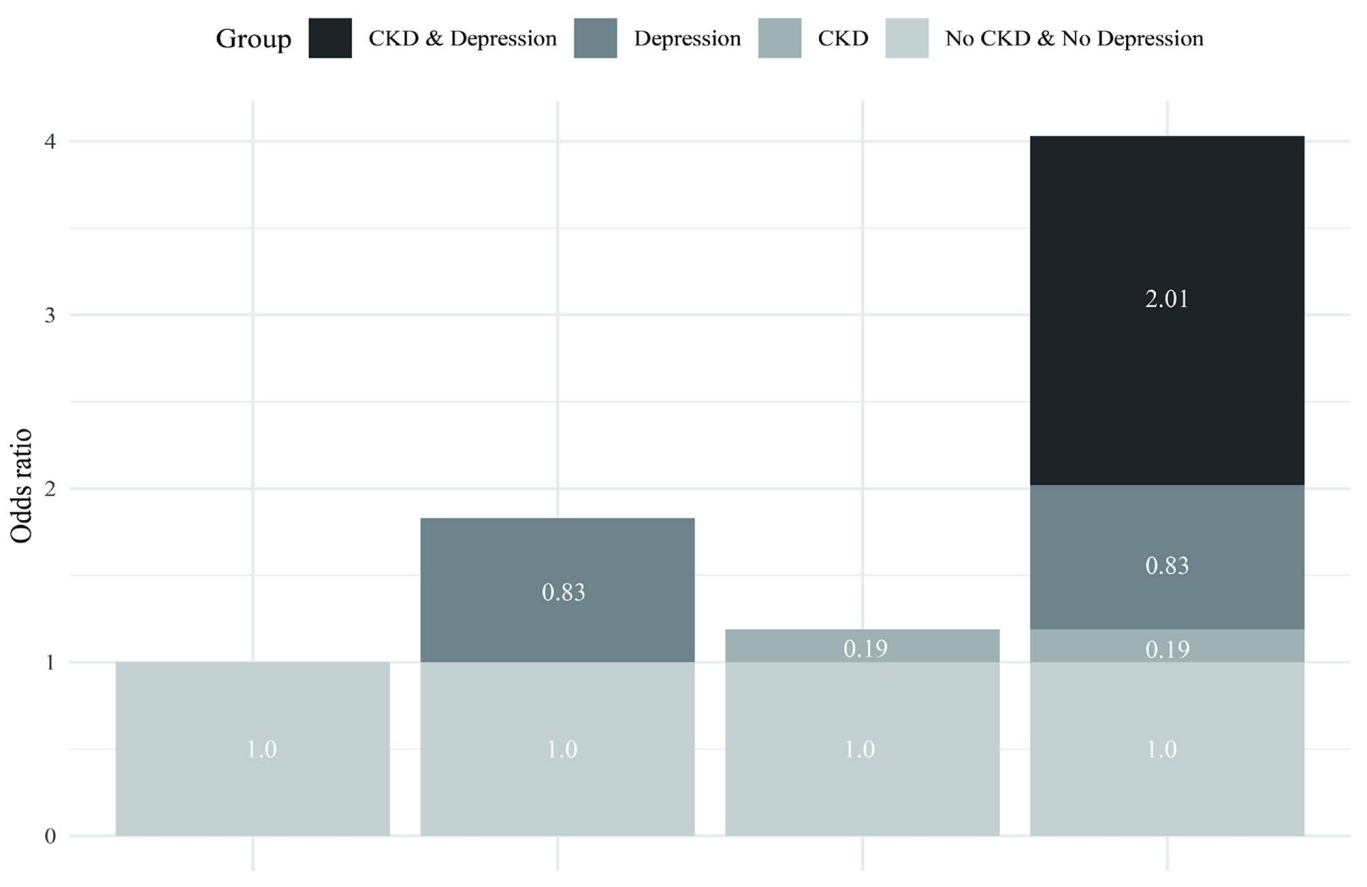
Interaction between CKD and depression on cognitive impairment in Model 3. Model 3: adjustment for age, race, education, annual family income, BMI, marital status, work activity, alcohol user, hypertension, diabetes, CVD. Abbreviations: CKD, chronic kidney disease; CVD, cardiovascular diseases

The findings presented in Table 2 indicate that in Model 3, CKD and depression had a significant synergistic effect on CoI (adjusted RERI = 2.01, 95% CI = 0.31–3.71; adjusted AP = 0.50, 95% CI = 0.25–0.75; adjusted S = 2.97, 95% CI = 1.27–6.92). The AP value of 0.50 in Model 3 suggests that 50% of CoI cases in the study sample were attributable to the interaction between CKD and depression. Figure 3 illustrates the additive interaction between CKD and depression in patients with CoI.

**Table 2:** Analysis of the interactive effect of CKD and depression

CKD	Depression	CoI/Total (n)	Model 1			Model 2			Model 3		
			OR	95% CI	P	OR	95% CI	P	OR	95% CI	P
0	0	316/1,575	Ref			Ref			Ref		
0 1 1	1 0 1	164/487 137/431 83/173	2.24 2.37 6.38	1.67–2.98 1.79–3.12 4.47–9.07	**< 0.001** **< 0.001** **< 0.001**	2.17 1.50 5.15	1.57–2.99 1.10–2.05 3.45–7.68	**< 0.001** **0.011** **< 0.001**	1.83 1.19 4.03	1.29–2.59 0.84–1.68 2.59–6.25	**0.001** 0.3 **< 0.001**
RERI (95% CI)			2.77 (0.60–4.94)			2.48 (0.49–4.47)			2.01 (0.31–3.71)		
AP (95% CI)			0.43 (0.22–0.65)			0.48 (0.25–0.71)			0.50 (0.25–0.75)		
S (95% CI)			2.06 (1.27–3.34)			2.48 (1.32–4.66)			2.97 (1.27–6.92)		

Model 1: unadjusted model; Model 2: adjustment for age and race; Model 3: adjustment for age, race, education, annual family income, BMI, marital status, work activity, alcohol user, hypertension, diabetes, CVD. Abbreviations: RERI, relative excess risk due to interaction; AP, attributable proportion of interaction; S, synergy index; CKD, Chronic kidney disease; CVD, Cardiovascular disease; OR, odds ratio; CI, confidence interval; CoI, cognitive impairment

### Effect of the interaction between CKD and depression severity on CoI

Only in Model 1, moderate-to-severe depression remained a significant variable, with a crude RERI of 5.56 (95% CI: 0.15–10.97), AP of 0.57 (95% CI: 0.31–0.83), and S of 2.79 (1.36–5.72). These results suggest a synergistic interaction between moderate-to-severe depression and CKD in patients with CoI (Table 3).

**Table 3:** Analysis of the interactive effects of CKD and depression severity

		Mild			Moderate-to-severe		
CKD	Depression	OR	95% CI	P	OR	95% CI	P
0	0	Ref			Ref		
0 1 1	1 0 1	1.98 2.37 5.14	1.38–2.79 1.79–3.12 3.34–7.83	**< 0.001** **< 0.001** **< 0.001**	2.73 2.37 9.66	1.79–4.08 1.79–3.12 5.50–17.1	**< 0.001** **< 0.001** **< 0.001**
RERI (95% CI) AP (95% CI) S (95% CI)		1.79 (-0.40–3.99) 0.35 (0.04–0.65) 1.76 (0.96–3.24)			5.56 (0.15–10.97) 0.57 (0.31–0.83) 2.79 (1.36–5.72)		

Abbreviations: RERI, relative excess risk due to interaction; AP, attributable proportion of interaction; S, synergy index; CKD, Chronic kidney disease; OR, odds ratio; CI, confidence interval

### Effect of interaction between depression and CKD severity on CoI

The interaction between depression and the severity of CKD did not result in significant impacts on CoI in all models. The results of the interactions are presented in Supplementary Table 1.

### Subgroup analysis

Across all models in the male subgroup, there was no detected interaction effect between depression and CKD on CoI (Supplementary Table 2). Within the female subgroup, an interaction was only observed in Model 1, while it was absent in both Model 2 and Model 3 (Supplementary Table 3).

Across all models in the obese (BMI ≥ 30 kg/m^2^) subgroup, there was no detected interaction between depression and CKD on CoI (Supplementary Table 4). Within the non-obese (BMI < 30 kg/m^2^) subgroup, Models 1 and 2 indicated an interaction, whereas Model 3 did not (Supplementary Table 5).

## Discussion

To investigate the potential interactions between depression and CKD and their effects on CoI, we analyzed the NHANES 2011–2014 data for individuals aged ≥ 60 years. Our findings revealed that among this population, 16.00% had CoI, 22.00% had CKD, and 21.12% experienced depression (with 13.67% having mild depression and 7.45% having moderate-to-severe depression). A significant synergistic effect was observed on CoI when depression and CKD coexisted in patients, particularly those with moderate-to-severe depression. Conversely, the severity of CKD did not demonstrate a similar impact. Appropriate data weighting was applied to ensure the accuracy and representativeness of our findings, and multiple models were established to adjust for potential confounding factors. This study provides novel insights by investigating the impact of the coexistence of depression and CKD on CoI, contributing to further research in this field.

Our study adjusted for various factors, including age, race, education, annual family income, marital status, work activity, alcohol use, hypertension, diabetes, and cardiovascular disease. The study sample found a significant positive correlation between CKD and CoI. This finding is consistent with a previously reported prospective study that observed a correlation between declining kidney function and CoI, even in patients with mild CKD [36]. Another large-scale national study in the United States revealed that CKD is associated with an increased prevalence of CoI, which rises by 11% for every 10 mL/min/1.73 m^2^ (< 60 mL/min/1.73 m^2^) decrease in eGFR [37]. Furthermore, studies have reported microstructural brain damage in end-stage kidney disease patients with MCI [38]. Some kidney-related metabolic markers have also been implicated in CoI [39]. Additionally, research by Cho et al. [40] indicates that albuminuria is associated with cortical thinning, primarily in the frontal and occipital lobes, and that it is correlated with an increase in white matter hyperintensity (WMH) load. Frontal lobe cortical atrophy is potentially mediated by WMH burden. CoI is commonly observed in patients with CKD, and various mechanisms, including vascular damage, uremic toxicity, oxidative stress, and peripheral/central inflammatory responses, might damage multiple cortical areas and subcortical neurons, leading to brain dysfunction and subsequent CoI [41–44]. Therefore, CKD is recognized as one of the strongest risk factors for CoI [45].

In the fully adjusted model, we found a significant correlation between depression and CoI. Some studies have indicated that individuals could experience CoI during the initial episode of depression. Furthermore, patients with recurrent episodes of depression might be at a greater risk of developing CoI compared to those who experience a single episode [20]. Persistent cognitive decline persists even in patients receiving effective drug treatment for depression [46, 47]. In the older population, depression is an important factor influencing cognitive decline [48]. Among older people with moderate-to-severe depression, CoI could be a strong predictor of dementia [49, 50]. Structural magnetic resonance imaging studies examining CoI and depression have shown volume reduction in various brain regions, including the insula, superior temporal gyrus (STG), inferior frontal gyrus, amygdala, hippocampus, thalamus, and cingulate gyrus. Shared volume reduction in the insula and STG might reflect communication difficulties and reduced involvement in mental and social stimulus activities, considered risk factors for CoI and major depressive disorder (MDD). These changes are more frequently observed in individuals with MDD [51–54]. WMHs are a potential cause of increased neuropsychiatric symptoms such as depression and can predict future changes in neuropsychiatric questionnaire responses. WMHs in the temporal and frontal lobes are particularly associated with these changes [55]. At the same time, WMHs in patients with MCI have been related to different cognitive functions (such as attention, executive function, and processing speed) [26]. Puzo C et al. suggested that WMHs might indicate an accelerated decline in cognitive and neuropsychiatric function, as evidenced by increased clinical dementia ratings and depression scores [27]. In summary, some scholars have found that multidimensional CoI exist both during and between episodes of depression. Depression and CoI share some common changes in brain structure, suggesting that depression may be a contributing factor to CoI. The mechanisms involved in this process include neuroinflammation, endocrine disorders, and abnormalities in neurotransmitter release [56, 57].

In addition to CoI, CKD leads to emotional changes, frequently leading to depression among patients with CKD; these individuals exhibit comparable changes in regional cerebral blood flow to those with mood disorders [58]. Inflammatory responses and cerebrovascular diseases are factors influencing depression in patients with CKD [45]. Inflammatory cytokines, hypothalamic-pituitary-adrenal (HPA) axis dysregulation, and oxidative stress have been implicated in the pathogenesis of depression [59]. CKD is a complex condition characterized by chronic inflammation, oxidative stress, and aging vascular and cellular damage [60]. These mechanisms might serve as the pathophysiological links between CKD and concurrent depression. The endothelial function of cerebral arteries is impaired during CKD [61], and the resulting vascular damage could cause brain white matter damage, cerebral infarction, or hemorrhage, leading to depression or CoI [45].

Previous studies have established CKD and depression as risk factors for CoI in older adults. Patients with CoI resulting from CKD or depression show reductions in the brain cortex and changes in white matter, although the underlying mechanisms are complex. Our research suggests that the combined impact of CKD and depression significantly increases the risk of CoI, compared to either condition occurring individually. The reasons for this increased risk and the underlying mechanisms are not yet fully understood; however, it is likely multifactorial.

Firstly, CKD and depression patients with CoI experience chronic stress and inflammation processes, and their combined action might intensify neuroinflammatory responses, further compromising cognitive function. Long-term exposure to an environment with elevated inflammatory cytokines could lead to depression and neuropsychiatric disorders [62] by activating brain inflammation pathways, leading to changes in neurotransmitter metabolism, neuroendocrine function, and neuronal plasticity [63]. Research by Hayley S et al. proposed that the combined effects of chronic stress and inflammation increase cytokine production and brain permeability, damaging vessels and brain function. These effects further lead to the activation of microglial cells, white matter damage, and the loss of neurons and glial cells. Patients initially present with either depression or MCI but eventually progress to dementia [64]. The pathogenesis of kidney and brain diseases involves oxidative stress and chronic-inflammatory processes, leading to a high incidence of neuropsychiatric disorders, CoI, and dementia among patients with CKD [65]. In patients with CKD, the loss of genes essential for maintaining the dynamic balance and neurogenesis of neural progenitor cells (NPCs) could result in learning and memory disorders. Moreover, the increased levels of inflammatory cytokines and free radicals in these patients could inhibit NPC proliferation and differentiation [12].

Secondly, when CKD and depression coexist, they further exacerbate problems such as cerebral hypoxia-ischemia and cerebrovascular lesions, increasing the risk of CoI in patients. Cerebrovascular diseases, including cerebral infarction, cerebral microhemorrhage, and brain white matter lesions, are common among patients with CKD [58]. These diseases are influenced by vascular factors, alterations in cerebral blood flow, and reductions in eGFR [66], leading to structural and functional damage in the brain, thereby affecting cognitive function. Existing research has demonstrated that vascular damage, impaired cerebral hemodynamics, and changes in the extracellular environment are the primary mechanisms underlying CoI in CKD [41]. Furthermore, cerebrovascular diseases and the onset of depression are closely associated. Stroke increases the risk of post-acute depression, with approximately one-third of patients with stroke experiencing depression [67]. The prevalence of post-stroke depression is on the rise [68]. The “vascular depression” hypothesis proposed that cerebrovascular disease or vascular risk factors could induce late-life depression syndrome, as evident from the frequent coexistence of this syndrome with cerebrovascular disease in the older population. Moreover, the vascular load is associated with vascular depression and cognitive deficits. Vascular depression is characterized by reduced integrity of white matter, executive dysfunction, functional disability, and a poor response to antidepressant treatment [69]. Arteriosclerosis and inflammatory responses could contribute to insufficient brain perfusion, triggering ischemic injury, disrupting neural connections, damaging the frontal lobe and other crucial neuronal networks, causing cortical and white matter lesions, and promoting or exacerbating depression and CoI [70, 71]. Moreover, factors like uremic toxins and hyponatremia could exacerbate endothelial dysfunction and arteriosclerosis [72].

Additionally, we hypothesize that certain genes might play a role in this process. Alpha-Klotho is associated with renal function decline, along with a potential role in the pathogenesis of CoI and depression. It could establish a neurobiological connection between CKD, depression, and dementia by regulating oxidative stress and inflammation [73]. Studies have indicated a correlation between decreased serum α-Klotho and cognitive decline in older adults [74, 75]. The Klotho gene (*KL*), predominantly expressed in the kidneys and choroid plexus of the brain, encodes the α-Klotho protein. Deficiency in α-Klotho could lead to cellular apoptosis caused by various cellular damage, including oxidative stress and defective autophagy and angiogenesis, that promotes renal fibrosis [76]. The expression of α-Klotho decreases early in the progression of CKD and is considered an early sensitive indicator of renal function decline. Its deficiency is a risk factor for CKD progression and the development of extrarenal complications [76]. Some research suggests that α-Klotho might influence the 5-HT neuronal development and contribute to late-life depression [77]. Recent studies have revealed a correlation between reduced serum α-Klotho levels and an increased prevalence of depression among middle-aged and older women [78].

Our findings demonstrate that the risk of developing CoI in patients is substantially elevated when CKD and depression are present together, compared to their separate effects, highlighting the critical need for clinicians to conduct thorough assessments in treating CoI patients. The results advance the formulation of individualized treatment strategies, encompassing drug therapy, psychological assistance, and lifestyle adjustments, to integratively manage CKD and depression, thus enhancing the cognitive state and life quality of patients. This finding offers specific practice guidance to clinicians, contributing to the enhancement of medical service outcomes and quality for this group of patients.

This study has a few limitations: Firstly, it relied on data from only two NHANES cycles, resulting in a relatively small sample size. Additionally, the sample was limited to participants from the United States. Future research should incorporate larger and more diverse samples from multiple centers. Secondly, the study design was cross-sectional, which precludes the exploration of causal relationships. Although we attempted to analyze the potential mechanisms of interaction, our data do not confirm whether these mechanisms actually played a role, necessitating further research for exploration.

## Conclusion

Our study results indicate a synergistic effect of CKD and depression on CoI. Subgroup analysis demonstrated a significant synergistic effect between moderate-to-severe depression and CKD on CoI, while no interaction was observed between depression and the severity of CKD on CoI. Our study provides epidemiological evidence for the mutual effects of concurrent CKD and depression on CoI. In clinical diagnosis and treatment, healthcare practitioners should consider the impact of these coexisting conditions on patients. Given the limited effectiveness of CoI treatment, addressing CKD and depression might enhance the therapeutic outcomes and prevent disease progression. Further research is warranted to explore the potential pathophysiological mechanisms underlying the synergistic effect of CKD and depression on CoI and the impact specific to different stages of CKD. Such investigations could provide clinicians with more targeted recommendations and assist patients in achieving better outcomes.

### Electronic supplementary material

Below is the link to the electronic supplementary material.

**Supplementary Material 1:** Supplementary data

## Data Availability

The datasets supporting the conclusions of this article are freely available at https://www.cdc.gov/nchs/nhanes.
