# Supplementary material for: Association of cognitive impairment with the interaction between chronic kidney disease and depression: findings from NHANES 2011–2014
Source: BMC Psychiatry. 2024 Apr 24;24:312. doi: 10.1186/s12888-024-05769-1 (PMC11044494; doi:10.1186/s12888-024-05769-1)
Supplement: Supplementary file 1 — Supplementary Material 1: Supplementary data [file 12888_2024_5769_MOESM1_ESM.docx]

**Supplementary Table 1. Analysis of the interactive effect of depression and CKD severity.**

|  |  | Non-progressive CKD | | | Progressive CKD | | |
| --- | --- | --- | --- | --- | --- | --- | --- |
| CKD | Depression | OR | 95% CI | P | OR | 95% CI | P |
| 0 | 0 | Ref |  |  | Ref |  |  |
| 0  1  1 | 1  0  1 | 2.24  2.13  4.82 | 1.67–2.98  1.59–2.83  3.26–7.05 | **<0.001**  **<0.001**  **<0.001** | 2.24  9.38  46.0 | 1.67–2.98  4.11–21.6  16.1–178 | **<0.001**  **<0.001**  **<0.001** |
| RERI (95% CI)  AP (95% CI)  S (95% CI) | | 1.45 (-0.40–3.30)  0.30 (0.01–0.59)  1.61 (0.93–2.80) | | | 35.34 (-18.43–89.11)  0.77 (0.46–1.08)  4.67 (1.33–19.3) | | |

RERI, relative excess risk due to interaction; AP, attributable proportion of interaction; S, synergy index; CKD, Chronic kidney disease; OR, odds ratio; CI, confidence interval.

**Supplementary Table 2. Analysis of the interactive effect of CKD and depression in male subgroup.**

| CKD | Depression | CoI/Total (n) | Model 1 | | | Model 2 | | | Model 3 | | |
| --- | --- | --- | --- | --- | --- | --- | --- | --- | --- | --- | --- |
|  |  |  | OR | 95% CI | P | OR | 95% CI | P | OR | 95% CI | P |
| 0 | 0 | 214/842 | Ref |  |  | Ref |  |  | Ref |  |  |
| 0  1  1 | 1  0  1 | 63/173  73/213  38/75 | 2.22  1.84  4.66 | 1.39–3.46  1.22–2.74  2.69–7.95 | **<0.001**  **0.003**  **<0.001** | 2.32  0.97  4.28 | 1.37–3.88  0.61–1.54  2.31–7.85 | **0.002**  **>0.9**  **<0.001** | 1.92  0.82  3.59 | 1.07–3.37  0.49–1.36  1.81–7.02 | **0.025**  0.5  **<0.001** |
| RERI (95% CI) | | | 1.60 (-0.97–4.17) | | | 1.99(-0.70–4.68) | | | -1.08 (-2.18–0.02) | | |
| AP (95% CI) | | | 0.34 (-0.06–0.75) | | | 0.46 (0.06–0.87) | | | -1.32 (-2.94–0.31) | | |
| S (95% CI) | | | 1.78 (0.77–4.13) | | | 2.54(0.78–8.28) | | | -0.20(NA) | | |

RERI, relative excess risk due to interaction; AP, attributable proportion of interaction; S, synergy index; CKD, Chronic kidney disease; CVD, Cardiovascular disease; OR, odds ratio; CI, confidence interval; CoI, cognitive impairment

Model 1: unadjusted model

Model 2: adjustment for age and race

Model 3: adjustment for age, race, education, annual family income, BMI, marital status, work activity, alcohol user, hypertension, diabetes, CVD

**Supplementary Table 3. Analysis of the interactive effect of CKD and depression in female subgroup.**

| CKD | Depression | CoI/Total (n) | Model 1 | | | Model 2 | | | Model 3 | | |
| --- | --- | --- | --- | --- | --- | --- | --- | --- | --- | --- | --- |
|  |  |  | OR | 95% CI | P | OR | 95% CI | P | OR | 95% CI | P |
| 0 | 0 | 102/733 | Ref |  |  | Ref |  |  | Ref |  |  |
| 0  1  1 | 1  0  1 | 213/314  64/218  53/98 | 2.58  3.14  8.77 | 1.75–3.81  2.12–4.63  5.43–14.2 | **<0.001**  **<0.001**  **<0.001** | 2.57  2.32  6.72 | 1.66–3.96  1.50–3.59  3.92–11.5 | **<0.001**  **<0.001**  **<0.001** | 2.18  1.59  4.61 | 1.36–3.48  0.98–2.57  2.51–8.47 | **0.001**  0.060  **<0.001** |
| RERI (95% CI) | | | 4.05 (0.18–7.91) | | | 2.83(-0.53–6.19) | | | -0.56 (-1.61–0.48) | | |
| AP (95% CI) | | | 0.46 (0.20–0.72) | | | 0.42 (0.10–0.74) | | | -0.35(-1.10–0.40) | | |
| S (95% CI) | | | 2.09 (1.17–3.71) | | | 1.98(1.00–3.93) | | | 0.51(0.14-1.88) | | |

RERI, relative excess risk due to interaction; AP, attributable proportion of interaction; S, synergy index; CKD, Chronic kidney disease; CVD, Cardiovascular disease; OR, odds ratio; CI, confidence interval; CoI, cognitive impairment

Model 1: unadjusted model

Model 2: adjustment for age and race

Model 3: adjustment for age, race, education, annual family income, BMI, marital status, BMI, work activity, alcohol user, hypertension, diabetes, CVD

**Supplementary Table 4. Analysis of the interactive effect of CKD and depression in obese subgroup.**

| CKD | Depression | CoI/Total (n) | Model 1 | | | Model 2 | | | Model 3 | | |
| --- | --- | --- | --- | --- | --- | --- | --- | --- | --- | --- | --- |
|  |  |  | OR | 95% CI | P | OR | 95% CI | P | OR | 95% CI | P |
| 0 | 0 | 94/533 | Ref |  |  | Ref |  |  | Ref |  |  |
| 0  1  1 | 1  0  1 | 71/222  50/157  39/93 | 2.28  3.14  8.77 | 1.42–3.64  1.52–4.02  2.88–8.53 | **<0.001**  **<0.001**  **<0.001** | 2.22  1.85  5.02 | 1.31–3.74  1.07–3.19  2.71–9.22 | **<0.001**  **<0.001**  **<0.001** | 1.61  1.36  3.26 | 0.90–2.85  0.73–2.52  1.67–6.31 | 0.11  0.3  **<0.001** |
| RERI (95% CI) | | | 1.22 (-1.40–3.83) | | | 1.94(-0.94–4.83) | | | -0.27 (-1.30–0.76) | | |
| AP (95% CI) | | | 0.24 (-0.19–0.68) | | | 0.39 (-0.02–0.79) | | | -0.20(-1.02–0.63) | | |
| S (95% CI) | | | 1.44(0.68–3.05) | | | 1.94(0.79–4.75) | | | 0.58(0.06-2.51) | | |

RERI, relative excess risk due to interaction; AP, attributable proportion of interaction; S, synergy index; CKD, Chronic kidney disease; CVD, Cardiovascular disease; OR, odds ratio; CI, confidence interval; CoI, cognitive impairment

Model 1: unadjusted model

Model 2: adjustment for age and race

Model 3: adjustment for age, race, education, annual family income, marital status, work activity, alcohol user, hypertension, diabetes, CVD

**Supplementary Table 5. Analysis of the interactive effect of CKD and depression in non-obese subgroup.**

| CKD | Depression | CoI/Total (n) | Model 1 | | | Model 2 | | | Model 3 | | |
| --- | --- | --- | --- | --- | --- | --- | --- | --- | --- | --- | --- |
|  |  |  | OR | 95% CI | P | OR | 95% CI | P | OR | 95% CI | P |
| 0 | 0 | 222/1042 | Ref |  |  | Ref |  |  | Ref |  |  |
| 0  1  1 | 1  0  1 | 93/265  87/187  44/80 | 2.35  2.35  9.23 | 1.61–3.38  1.67–3.28  5.64–15.2 | **<0.001**  **<0.001**  **<0.001** | 2.19  1.37  5.85 | 1.43–3.31  0.93–2.01  3.36–10.20 | **<0.001**  0.11  **<0.001** | 1.94  1.17  4.48 | 1.23–3.05  0.76–1.78  2.41–8.34 | **0.004**  0.5  **<0.001** |
| RERI (95% CI) | | | 5.54 1.12–9.96) | | | 3.29(0.12–6.47) | | | -0.27 (-1.30–0.76) | | |
| AP (95% CI) | | | 0.60 (0.39–0.81) | | | 0.56 (0.29–0.84) | | | -0.20(-1.02–0.63) | | |
| S (95% CI) | | | 3.05(1.61–5.81) | | | 3.11(1.30–7.43) | | | 0.58(0.06-2.51) | | |

RERI, relative excess risk due to interaction; AP, attributable proportion of interaction; S, synergy index; CKD, Chronic kidney disease; CVD, Cardiovascular disease; OR, odds ratio; CI, confidence interval; CoI, cognitive impairment

Model 1: unadjusted model

Model 2: adjustment for age and race

Model 3: adjustment for age, race, education, annual family income, marital status, work activity, alcohol user, hypertension, diabetes, CVD
